# Distress-Mediated Remodeling of Cardiac Connexin-43 in a Novel Cell Model for Arrhythmogenic Heart Diseases

**DOI:** 10.3390/ijms231710174

**Published:** 2022-09-05

**Authors:** Carl-Mattheis Wahl, Constanze Schmidt, Markus Hecker, Nina D. Ullrich

**Affiliations:** 1 Institute of Physiology and Pathophysiology, Division of Cardiovascular Physiology, Heidelberg University, 69120 Heidelberg, Germany; 2 Department of Cardiology, Angiology and Pneumology, University Hospital Heidelberg, 69120 Heidelberg, Germany; 3 DZHK (German Center for Cardiovascular Research), Partner Site Heidelberg/Mannheim, Heidelberg University, 69120 Heidelberg, Germany

**Keywords:** Cx43, iPSC-cardiomyocytes, chronic oxidative distress, miR-1, tachypacing

## Abstract

Gap junctions and their expression pattern are essential to robust function of intercellular communication and electrical propagation in cardiomyocytes. In healthy myocytes, the main cardiac gap junction protein connexin-43 (Cx43) is located at the intercalated disc providing a clear direction of signal spreading across the cardiac tissue. Dislocation of Cx43 to lateral membranes has been detected in numerous cardiac diseases leading to slowed conduction and high propensity for the development of arrhythmias. At the cellular level, arrhythmogenic diseases are associated with elevated levels of oxidative distress and gap junction remodeling affecting especially the amount and sarcolemmal distribution of Cx43 expression. So far, a mechanistic link between sustained oxidative distress and altered Cx43 expression has not yet been identified. Here, we propose a novel cell model based on murine induced-pluripotent stem cell-derived cardiomyocytes to investigate subcellular signaling pathways linking cardiomyocyte distress with gap junction remodeling. We tested the new hypothesis that chronic distress, induced by rapid pacing, leads to increased reactive oxygen species, which promotes expression of a micro-RNA, miR-1, specific for the control of Cx43. Our data demonstrate that Cx43 expression is highly sensitive to oxidative distress, leading to reduced expression. This effect can be efficiently prevented by the glutathione peroxidase mimetic ebselen. Moreover, Cx43 expression is tightly regulated by miR-1, which is activated by tachypacing-induced oxidative distress. In light of the high arrhythmogenic potential of altered Cx43 expression, we propose miR-1 as a novel target for pharmacological interventions to prevent the maladaptive remodeling processes during chronic distress in the heart.

## 1. Introduction

Arrhythmogenic cardiac diseases comprise a large group of diseases characterized by irregular electrical activity of the heart with severe consequences for the contractile behavior of the cardiac muscle and thus for ejection fraction. Besides a multitude of mutations affecting different ion channels and their functions, there are many more reasons and acquired conditions that can lead to the development of arrhythmias, comprising systemic diseases such as diabetes, hypertension or the natural process of ageing. However, in principle, the diverse diseases converge at the cellular level and lead to dysfunction of the cell-to-cell junctions, and as a consequence, to alterations of electrical signal propagation across the cardiac muscle [1,2,3,4,5,6].

A special feature of cardiac tissue is the formation of a functional syncytium with one key component of intercellular junctions being gap junctions. These intercellular tunnels are built from two hemichannels or connexons built from connexins. Connexins are transmembrane proteins consisting of four membrane spanning domains and assemble as hexamers in the membrane to form intercellular channels responsible for electrical signal propagation across the cardiac tissue. Therefore, they denote the mechanistic basis of a functional syncytium. In ventricular cardiomyocytes, the predominant gap junction protein is connexin-43 (Cx43), although other isoforms coexist at different levels of expression with different conduction properties [7,8,9].

In healthy cardiomyocytes, Cx43 expression is preferentially directed towards intercalated discs where gap junctions are formed, and this special localization ensures a specific direction of signal spreading along the long axis of cardiomyocytes and cardiac tissue [10]. However, in the diseased heart, it has been shown that Cx43 expression is not only reduced but also dislocated to lateral membrane areas of the cardiomyocytes [11], thereby changing the dynamics and direction of electrical signal spreading. In addition, this lateralization may lead to critical changes in conduction properties of the affected cardiac tissue. Decreased focal conduction velocity represents a high risk for the development of re-entry circuits ultimately resulting in tachyarrhythmias and fibrillation.

Up until now, the triggers and mechanisms underlying these critical remodeling processes remain to be elucidated. It has been suggested that stressful stimuli may harm single cells resulting in local downregulation of gap junctions. This may also be interpreted as a protective mechanism to avoid harmful metabolites or cell signals to be spread to neighboring cells [12]. However, during chronic stress, protective mechanisms may tip and lead to long term damage of the affected tissue. Stressful stimuli can be of different characters, e.g., prolonged periods of tachycardia as observed in patients with high blood pressure or mental stress. At the cellular level, chronic stress is associated with inflammation and elevated levels of reactive oxygen species (ROS) [6].

In healthy cardiomyocytes, low amounts of ROS are constantly produced as byproducts of oxygen metabolism or cell signaling processes and controlled by multiple ROS scavenging enzymes, e.g., superoxide dismutase and glutathione (GSH) peroxidase, to keep a healthy equilibrium of cellular ROS levels [2]. In contrast, in disease or during ageing, accumulation of by-products of the oxygen metabolism may lead to a progressive imbalance in the redox equilibrium of the cardiomyocyte. Enhanced chronic oxidative stress is involved in maladaptive structural and functional remodeling and typically affects proteins of the intercalated disc (ICD) [13]. Furthermore, the negative consequences of sustained oxidative stress, also termed oxidative distress [14], strongly influence the Ca^2+^ handling proteins of the excitation-contraction coupling machinery, leading to an imbalance in diastolic Ca^2+^ levels and the facilitation of extrasystolic beats. Together with gap junction remodeling and altered electrical signal propagation across the myocardium, the probability of the development of arrhythmias is severely increased during chronic distress [2].

Up until now, the mechanistic sequence of maladaptive events leading from chronic cardiomyocyte stress to gap junction remodeling has not yet been elucidated. Therefore, this study has been designed to investigate the cellular processes that underlie Cx43 downregulation in periods of chronic cell stress. Since it is impossible to perform long term studies in adult cardiomyocytes due to culture-induced dedifferentiation processes, we turned to the novel cell model of murine induced pluripotent stem cell-derived ventricular cardiomyocytes (iPSC-CMs). In the past, we have intensively characterized gap junction function and intercellular communication in these cells and demonstrated that Cx43 expression occurs at very low levels compared to native cardiomyocytes [15,16]. At the same time, a higher Cx43 expression level can be achieved in these cells [9]. Therefore, these cells represent an optimal culture model to investigate control mechanisms of Cx43 expression [17]. Based on large animal studies [18,19,20] and established cell culture models to investigate mechanisms of arrhythmias [21], we applied chronic tachypacing as a stimulus to induce chronic stress in iPSC-CMs. We tested the hypothesis that persistent tachypacing leads to prolonged oxidative distress in these cardiomyocytes, which in turn affects Cx43 membrane expression. Furthermore, we postulate that the resulting buildup of ROS leads to long-term modifications of Cx43 expression by enhancing the expression of a specific micro-RNA, designated miR-1. This miRNA has not only been shown previously to control Cx43 protein expression [22], but there is also strong evidence for increased miR-1 expression in arrhythmogenic diseases and experimental models of arrhythmias [23,24,25]. Using a broad spectrum of different techniques, we follow the pathway of tachypacing-induced Cx43 remodeling in iPSC-CMs with the aim to identify novel cellular targets for the development of therapeutic agents to reduce the burden of arrhythmogenic diseases.

## 2. Results

### 2.1. Live Monitoring of ROS Production

The first set of experiments was conducted to evaluate the reliability of H_2_DCF-based monitoring of reactive oxygen species (ROS) production in the cardiac HL-1-cells and in induced pluripotent stem cell-derived ventricular cardiomyocytes (iPSC-CMs), both of murine origin for optimal comparison. In particular, attention was paid to general dynamics of the development of the fluorescence signal, which primarily stems from the rapid oxidation of H_2_DCF to DCF by H_2_O_2_, and to putative processes of autoxidation. The term ROS production in the following thus primarily refers to H_2_O_2_ formation in the cell although H_2_DCF can also be oxidized to DCF by other ROS such as hydroxyl radicals, (lipid) hydroperoxides and peroxynitrite, but with much lower efficacy. Notably, H_2_DCF cannot be oxidized to DCF by superoxide anions [26]. For better evaluation, the effect of the mild oxidant TBHP was tested during confocal imaging of HL-1-cells and iPSC-CMs.

**Figure 1:**
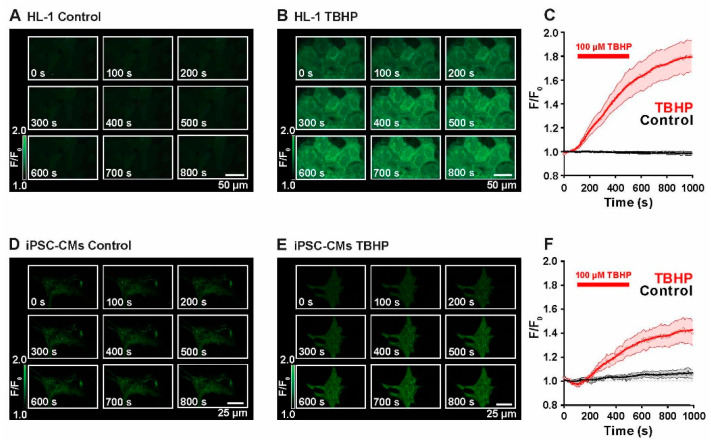
Live imaging of ROS production in HL-1 cells and iPSC-CMs at different conditions. (**A**) Single confocal images recorded during a time-course experiment of imaging ROS-dependent oxidation of H_2_DCF to DCF in unstimulated HL-1 cells. (**B**) Time-dependent increase in DCF fluorescence triggered by TBHP perfusion of HL-1 cells. (**C**) Time course of the average DCF fluorescence during imaging of control (black) and TBHP-treated (red) HL-1 cells (*n* = 3). (**D**) Single confocal images of unstimulated iPSC-CMs during a time course recording. (**E**) Time-dependent increase in DCF fluorescence triggered by TBHP perfusion of iPSC-CMs. (**F**) Time course of the average DCF fluorescence during imaging of control (black) and TBHP-treated (red) iPSC-CMs (*n* = 3). The red bar indicates the time of TBHP perfusion (*n* numbers of repeated experiments).

Figure 1A shows unstimulated HL-1 cells at control conditions during constant perfusion with Tyrode solution. During the entire imaging period (*t* = 1000 s), the DCF fluorescence signal remained stable, indicating constant levels of ROS and no detectable autoxidative activity of the dye. At 1000 s, normalized fluorescence corresponded to 1.0 ± 0.1 F/F_0_ (*n* = 3). To investigate dye behavior during live-imaging and to evaluate the response to an oxidizing stimulus, cells were perfused with 100 µM TBHP for a duration of 400 s. As displayed in Figure 1B, fluorescence intensity increased immediately after adding TBHP and ended in a plateau after cessation of perfusion. Average time courses of control and TBHP-perfused HL-1 cells are summarized in Figure 1C. This experiment demonstrates that DCF adequately monitors the rise in cellular ROS production. At *t* = 1000 s, fluorescence intensity was determined to be 1.8 ± 0.1 F/F_0_ (*n* = 3). Please note that the fluorescence signal in these confocal images arises from biochemical reactions in the cell in response to the externally applied oxidant. The steepness of the slope indicates the activity of ROS-producing enzymes in the cardiomyocytes. However, the increase in fluorescence is not reversible after washout of the oxidant but levels remain constantly elevated because the oxidation of H_2_DCF to DCF oxidation is not reversible. Figure 1D,F shows the same set of experiments performed in iPSC-CMs. Again, at control conditions, there was no significant increase in fluorescence intensity during the time of imaging (1.1 ± 0.04 at *t* = 1000 s, *n* = 3). Addition of TBHP strongly increased the fluorescence signal emitted by ROS-activated DCF (1.4 ± 0.1 at *t* = 1000 s, *n* = 3).

These experiments demonstrate that the fluorescent ROS indicator H_2_DCF gives reliable information about ROS production in HL-1 cells and iPSC-CMs, therefore, it was used in the next set of experiments to indicate intracellular ROS production upon different biological stimuli and conditions.

### 2.2. Electrical Stimulation and Tachypacing of iPSC-CMs

**Figure 2:**
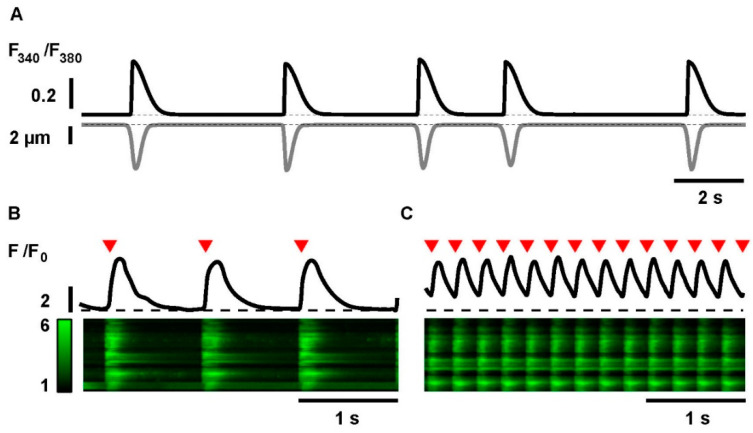
Sample traces of Ca^2+^ transients (black) and cell shortening (grey) in iPSC-CMs during spontaneous activity. (**A**) Ca^2+^ transients and contractions were simultaneously measured by fura-2 and edge detection using the IonOptix setup. (**B**) Sample line scan image and corresponding line profile (black) of iPSC-CMs during electrical stimulation at 1 Hz and tachypaced at 4 Hz (**C**). Ca^2+^ transients were measured with fluo-4 using a LSCM. Stimulation frequencies are indicated by red arrows in the top traces.

iPSC-CMs exhibit intrinsic spontaneous contractile activity, which usually occurs at a relatively low and irregular frequency. In this study, field stimulation was used to impose a defined beating rhythm at a basal frequency close to the natural beating frequency of these cells (1 Hz), and at high frequency as a tool to induce cellular stress (4 Hz, as previously established and reported by Yeh et al.) [21]. Three test groups were defined: no pacing, cells paced at 1 Hz, and tachypacing at 4 Hz. In order to validate the cell response to field stimulation, Ca^2+^ transients and contraction were simultaneously measured in iPSC-CMs. Figure 2A displays a sample trace of Ca^2+^ transients (in black) and the corresponding contractions, shown as cell shortening (in grey), during spontaneous activity. Figure 2B,C show sample line scan images and the corresponding line profiles of iPSC-CMs during pacing at 1 Hz and during tachypacing at 4 Hz. These data demonstrate that iPSC-CMs respond adequately to an imposed stimulation frequency and even adapt reliably to pacing frequencies up to 4 Hz. In order to investigate frequency-dependent effects and ROS-generation, the experiments were based on pacing at 1 Hz as control rhythm and at 4 Hz as electrical model for tachypacing.

### 2.3. Tachypacing as a Model to Induce Oxidative Distress in iPSC-CMs

**Figure 3:**
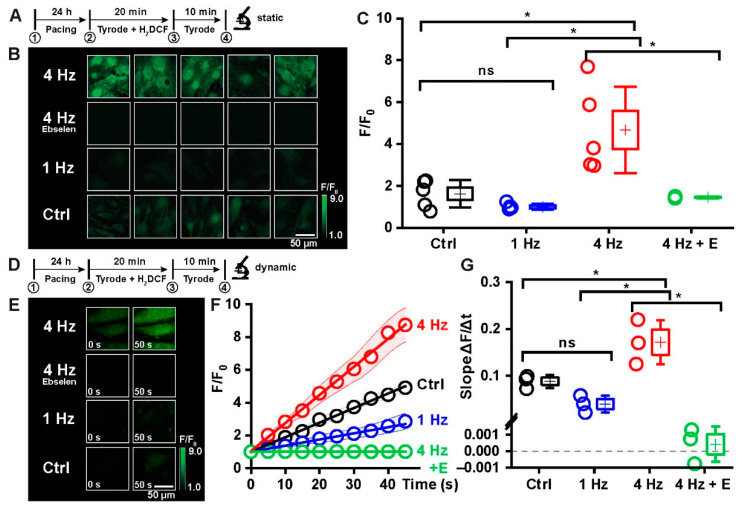
Tachypacing-induced ROS generation in iPSC-CMs. (**A**) Outline of the ROS imaging experiment indicating the four different phases. (**B**) Confocal images of cells loaded with the ROS-indicator H_2_DCF were taken immediately after pacing of the cells for 24 h. (**C**) Statistical analysis of the mean fluorescence intensities of all images and conditions recorded in phase 4a (Ctrl: *n* = 5, 1 Hz: *n* = 5, 4 Hz: *n* = 5, 4 Hz + ebselen, E: *n* = 3), normalized to Ctrl. (**D**) Experimental protocol. (**E**) Sample images taken at the beginning (*t* = 0) and end (*t* = 50 s) of ROS imaging. (**F**) Mean traces of the time-dependent increase in DCF fluorescence intensity after phase 4b. (**G**) Slope calculated from the data shown in (**F**) (*n* = 3 in all four conditions), normalized to Ctrl. Please note that a negative slope, as seen in one sample of the ebselen-treated cells, can be explained as a slight bleaching effect on the fluorescent dye during repetitive image acquisition. Please note: due to different acquisition modes, the fluorescence intensity cannot be compared between (**B**,**E**) (*n* numbers of repeated experiments; ns—no significant difference; * statistically significant difference).

In order to investigate the effect of rapid external stimulation on cellular responses to stress situations, both techniques were combined in the next set of experiments. The following experimental protocol was developed specifically to evaluate ROS production in response to different electrical stimulation frequencies in iPSC-CMs. The protocol consisted of four phases: Phase 1 comprised 24 h of either no pacing, pacing at 1 Hz or at 4 Hz. Phase 2: after this time, cells were loaded with the ROS-sensitive fluorescent indicator H_2_DCF diacetate for 20 min under the same experimental conditions (no pacing or pacing). In phase 3, fresh Tyrode solution was applied for 10 min to warrant full de-esterification of the H_2_DCF. In phase 4, confocal images were recorded: either static images (5 images per well, phase 4a, Figure 3B) or image series of 10 images in 50 s to monitor the dynamics of the increase in fluorescence intensity over time (phase 4b, Figure 3E).

Figure 3 summarizes the results of this experimental series. Figure 3A shows 5 sample images of each condition of phase 4a, while in Figure 3C, statistical evaluation of the fluorescence intensities is illustrated. They reveal the following: In control cells, the mean fluorescence intensity of the DCF signal corresponded to 1.6 ± 0.3 F/F_0_ (*n* = 5), in cells paced at 1 Hz, the DCF signal was close to baseline (1.0 ± 0.1 F/F_0_, *n* = 5), indicating no significant production of ROS under these conditions. In contrast, in cells paced at 4 Hz, fluorescence intensity increased to 4.7 ± 0.9 F/F_0_ (*n* = 5). Tachypacing thus induces a significant increase in ROS production in the iPSC-CMs. Incubation of cells paced at 4 Hz with the GSH peroxidase-like H_2_O_2_ scavenger ebselen (1 µM) during the entire experimental procedure significantly reduced the DCF fluorescence signal close to that of cells paced at 1 Hz (1.4 ± 0.03 F/F_0_, *n* = 3), suggesting that the tachypacing-induced rise in DCF fluorescence intensity is based on primary or secondary formation of H_2_O_2_ or lipid hydroperoxides in iPSC-CMs [26].

Figure 3D–G summarizes the data of phase 4b showing representative images taken at the beginning (t = 0 s) and at the end (t = 50 s) of the time series (Figure 3D) and the mean fluorescence intensities of the DCF signal of every image taken during the time series (Figure 3F). In Figure 3G, the slope of the rise in DCF fluorescence is summarized. These data demonstrate that tachypacing induced a strong and almost linear increase in ROS production over time with a slope of 0.17 ± 0.03 ΔF/s (*n* = 3) as compared to pacing at 1 Hz (0.04 ± 0.01 ΔF/s) or no pacing (0.09 ± 0.01 ΔF/s). In the presence of ebselen, the tachypaced cells essentially did not exhibit any increase in DCF fluorescence intensity (Figure 3E). Worth mentioning is that in control cells, the slope is slightly but not significantly steeper than in cells paced at 1 Hz. This could indicate that regular pacing at 1 Hz leads to healthier cell metabolism than the rather irregular spontaneous activity of untreated cells. We conclude from these results that chronic tachypacing elicits a significant rise in ROS production in iPSC-CMs that is due to primary or secondary formation of H_2_O_2_ or lipid hydroperoxides.

### 2.4. ROS-Mediated Cx43 Reduction via miR-1

**Figure 4:**
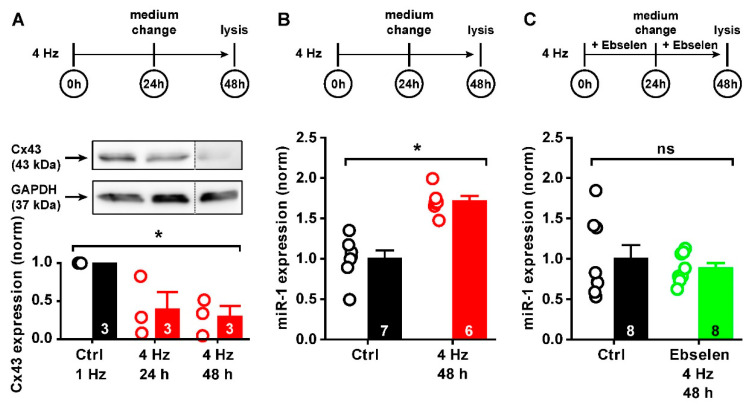
Effect of tachypacing on Cx43 expression and miR-1 levels in iPSC-CMs. Top traces show the experimental protocol. (**A**) Representative Western blot and analysis of total Cx43 protein abundance in control (Ctrl, 1 Hz) and tachypaced cells after 24 h and 48 h of electrical stimulation, respectively, with statistical analysis of Cx43 protein normalized to GAPDH and Ctrl. (**B**) Relative miR-1 expression by Ctrl and tachypaced iPSC-CMs after 48 h, normalized to Ctrl. (**C**) Same experiment as in (**B**) but performed in the presence of 1 μM ebselen (*n* numbers of repeated experiments are indicated in the columns; ns—no significant difference; * statistically significant difference).

In the next set of experiments, we tested the hypothesis that chronic distress induced by tachypacing influences Cx43 expression, which is mediated by oxidative distress, i.e., a relative increase in cellular ROS. In two experimental setups, iPSC-CMs were paced for the first 24 h, then for a prolonged time of 48 h at 1 Hz or 4 Hz. Afterwards, whole cell lysates were collected and Cx43 protein expression was determined. Figure 4A shows the experimental protocol in the upper trace together with a sample blot and the statistical evaluation of 3 independent experiments (uncropped blots are available in Supplemental Figure S1). After 24 h, a tendency of a reduction in Cx43 expression was observed, though not significantly different to control. However, the prolonged pacing time of 48 h revealed that compared to control cells paced at 1 Hz, tachypacing at 4 Hz significantly reduced Cx43 expression after 48 h (to 40 and 30% of control, respectively). Expression of the reference protein GAPDH showed similar expression levels in all conditions (*p* = 0.815). Since the effect of tachypacing on Cx43 expression was significant after 48 h, we continued with this stimulation protocol for the next experiments. These data demonstrate that in iPSC-CMs chronic tachypacing leads to a reduction in Cx43 expression and, therefore, this experimental setup serves as an excellent cell model to study the underlying mechanisms of controlling Cx43 expression in the situation of cell stress.

Knowing that micro-RNA 1 (miR-1) is an important modulator of Cx43 protein abundance, we investigated in the next step, whether chronic tachypacing and the pacing-induced increase in oxidative stress influences the expression levels of miR-1. Using the same protocol, miR-1 expression was assessed in iPSC-CMs after 48 h of tachypacing relative to control cells. Figure 4B summarizes the findings that miR-1 was expressed at significantly higher levels compared to control cells (by 70% relative to control cells paced at 1 Hz), and this effect was fully reversible using the H_2_O_2_/lipid hydroperoxide scavenger ebselen (1 µM) during the entire experimental procedure (−10% relative to control cells paced at 1 Hz, Figure 4C). Please note: since the strongest effect of miR-1 on Cx43 expression appeared after 48 h, miR-1 expression was likewise examined at this time point.

Taken together, these data demonstrate for the first time that prolonged periods of cellular stress induced by rapid pacing lead to oxidative distress, which directly influences and enhances miR-1 expression and thus reduces Cx43 protein abundance.

### 2.5. miR-1 Control of Cx43 Expression

**Figure 5:**
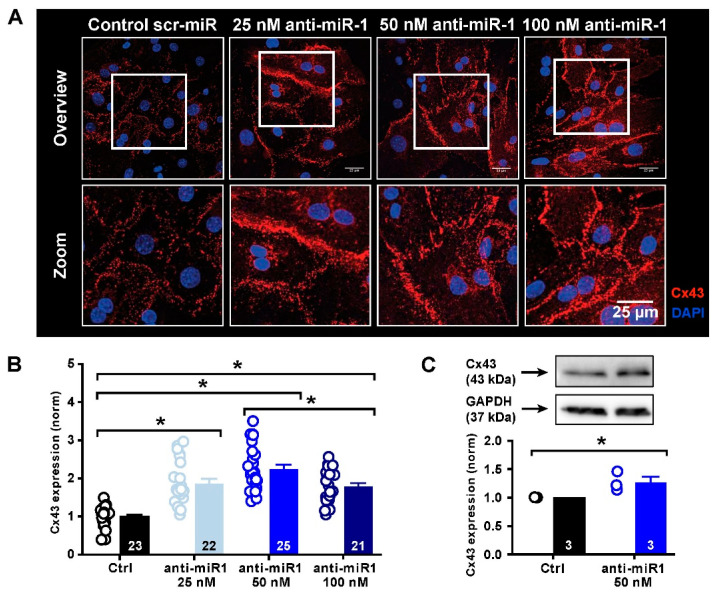
Effect of antimiR-1 on Cx43 expression in iPSC-CMs. (**A**) Representative confocal images of Cx43 immunofluorescence analysis in iPSC-CMs incubated with either scrambled control antimiR (scr-miR) or different concentrations of the miR-1-neutralizing antisense oligonucleotide (antimiR-1). (**B**) Statistical summary of the immunofluorescence analysis. Data are normalized to exposure to the scrambled control anti-miR (Ctrl) (*n* numbers of analyzed images of 3 individual experiments are indicated in the columns). (**C**) Representative Western blot and analysis of total Cx43 protein showing relative increase in total Cx43 protein expression by neutralization of miR-1 using 50 nM anti-miR-1. Data are normalized to GAPDH and Ctrl (*n* numbers of repeated experiments are indicated in the columns; * statistically significant difference).

Given the fact that tachypacing and chronic oxidative stress lead to an increase in miR-1 expression and a reduction in Cx43 protein expression, we next wanted to see if Cx43 expression is directly dependent on miR-1 activity. To this end, we functionally neutralized miR-1 using a specific oligonucleotide directed against miR-1, designated antimiR-1. iPSC-CMs were incubated with different concentrations of antimiR-1, and the expression of Cx43 was assessed by immunofluorescence and Western blot analysis. Figure 5A shows that incubation of iPSC-CMs with 25 nM, 50 nM and 100 nM of antimiR-1 significantly increased sarcolemmal Cx43 expression in all conditions compared to a scrambled control antimiR (Figure 5B). This effect peaked notably at 50 nM (140% relative to control) but was quite similar at 25 and 100 nM, which may be associated with the expression efficiency of the antisense oligonucleotide used. In Figure 5C, total Cx43 protein content was assessed by Western blot analysis in the presence of 50 nM antimiR-1 (uncropped blots are available in Supplemental Figure S2). These data confirm an overall significant increase in total Cx43 protein abundance upon miR-1 inhibition, which, however, was much less pronounced (30% relative to control) than that detected by immunofluorescence analysis. These differences can be explained by distinct Cx43 pools in the sarcolemma and in intracellular storage organelles [27].

These data demonstrate that Cx43 expression is strongly modulated by miR-1. Selective inhibition of miR-1 by antimiR-1 significantly increases Cx43 expression in iPSC-CMs, most importantly its abundance in the sarcolemma.

### 2.6. Functional Characterization of Intercellular Coupling by Modulation of Cx43 Expression in iPSC-CMs

**Figure 6:**
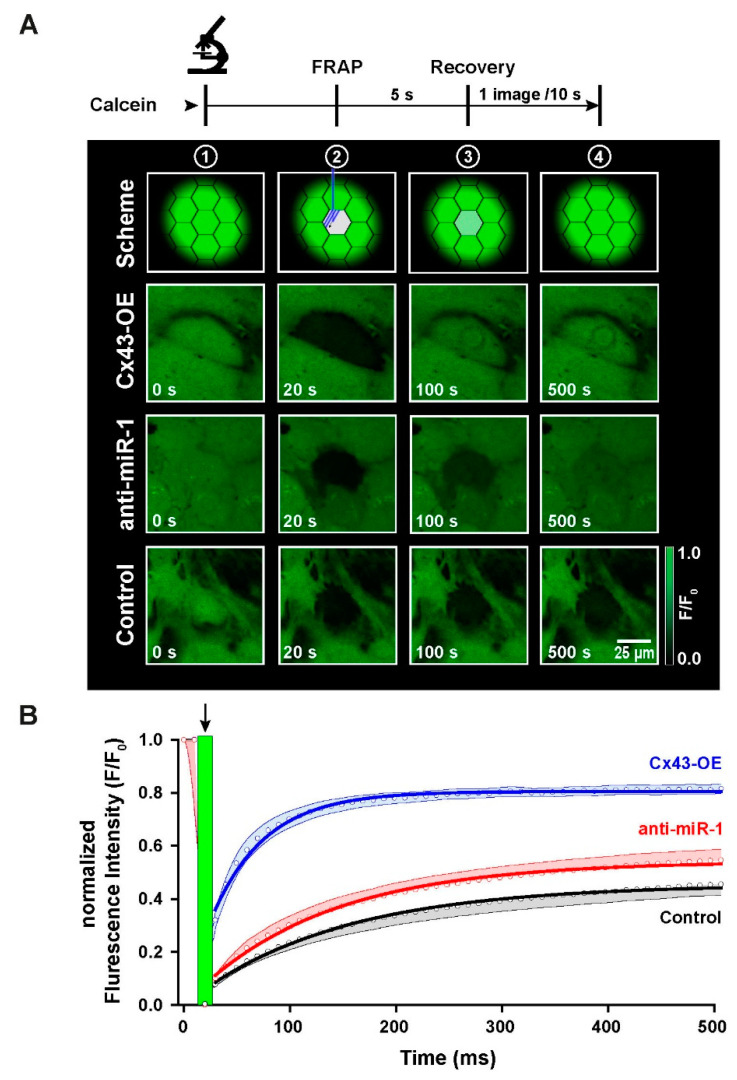
Fluorescence recovery after photobleaching (FRAP) of calcein in iPSC-CMs. (**A**) The upper trace and scheme show the experimental protocol, underneath representative confocal sample images are shown for scrambled control oligonucleotide-treated iPSC-CMs (Control), anti-miR-1-treated cells and Cx43-overexpressing cells (Cx43-OE). Images were recorded at 4 different time points during the recordings. (**B**) Time course of FRAP for the three different conditions. The green bar and arrow indicate the time of photobleaching (Control: *n* = 40, anti-miR-1: *n* = 31, Cx43-OE: *n* = 23; *n* measurements from 3 different cultures).

To test whether enhanced Cx43 protein expression by selective inhibition of miR-1 leads to formation of more functional gap junctions in iPSC-CMs, we next investigated the temporal properties of cell–cell coupling in FRAP experiments. For this set of experiments, iPSC-CMs were seeded at high density to form a closed monolayer, and then loaded with the gap junction permeant fluorescent dye calcein. One cell in the center of a tight group of cells was photobleached, and then the dynamics of recovery of fluorescence via dye diffusion through gap junctions with neighboring cells was monitored and recorded over time. This experiment consisted of 3 test groups: the first group was treated with the scrambled control anti-miR and served as control group, the second group was treated with antimiR-1, and the third group comprised Cx43-overexpressing cells (Cx43-OE) as a positive control to demonstrate the strong differences in dye diffusion kinetics when many functional gap junctions are present. Figure 6A shows the protocol of the experiment in the upper part. The confocal sample images were taken just before photobleaching (t = 0 s), directly after photobleaching (t = 20 s), during fluorescence recovery at t = 100 s and at t = 500 s to demonstrate the final level of FRAP. Figure 6B displays the FRAP traces during the time course of these experiments up to 500 s when the traces levelled off in a plateau phase. The recovery curves were fitted with an exponential function to extract the time constant of fluorescence recovery. While the control trace displayed the slowest recovery curve (τ = 102 s), levelling off at less than 50% above the initial fluorescence intensity, Cx43-OE cells exhibited a very rapid FRAP curve (τ = 36 s) reaching the new plateau already after about 100 s. Treatment of iPSC-CMs with the antisense oligonucleotide antimiR-1 also significantly accelerated the recovery curve (τ = 91 s) and resulted in a higher level of fluorescence recovery towards the end of the experimental phase compared to control cells.

These data demonstrate for the first time that miR-1 inhibition not only results in an enhanced Cx43 protein abundance in iPSC-CMs, notably in the sarcolemma, but also that these additional Cx43 proteins contribute to the formation of more functional gap junctions connecting the cells with each other.

## 3. Discussion

Cardiac diseases and heart failure are associated with chronic stress and altered connexin-43 (Cx43) protein expression in cardiomyocytes with the consequence of arrhythmogenic modifications in the heart. In this study, we have designed a new mouse experimental model employing murine induced pluripotent stem cell-derived ventricular cardiomyocytes (iPSC-CMs) to examine the molecular mechanisms underlying this observation. Our data demonstrate for the first time that pacing-induced chronic stress causes oxidative distress in iPSC-CMs as assessed by measuring intracellular reactive oxygen species (ROS) formation. In addition, we present the first experimental evidence that sustained oxidative stress is causally linked to the pathophysiological remodeling of gap junctions through their epigenetic control via micro-RNA 1 (miR-1) thereby altering the electrical properties of these cardiomyocytes.

### 3.1. Experimental Models to Induce Cell Stress in iPSC-CMs

The idea of using long-term tachypacing to induce arrhythmias arrived from experimental studies to specifically trigger heart failure by ventricular electrophysiological alterations in clinically relevant animal models [28,29]. Over the years, it has been shown that a large variety of cardiac pathologies leads to significant remodeling of cardiac ion channels and gap junctions resulting in severe alterations of action potential duration and progressive conduction slowing [30,31]. These modifications can be mimicked in animal models of pacing-induced tachycardia [32]. More recently, the HL-1 cell line has proven a valuable tool to investigate pacing-induced cellular modifications and electrical remodeling likewise in vitro [21,33]. Here, we have established and validated in vitro tachypacing in iPSC-CMs to investigate cell stress-dependent remodeling of Cx43 and gap junctions. In previous studies, we have intensively examined the functional properties of iPSC-CMs not only at the level of single cells or cell pairs, but also in the context of a functional syncytium [15,17,34,35]. These studies demonstrate that Cx43 expression is very small in these cells, leading to a relatively low amount of gap junctions compared to native cardiomyocytes of the same species [15]. This reduced intercellular coupling results in slow electrical signal propagation from cell to cell with the consequence of dyssynchronous electrical and contractile activities within multicellular preparations [16]. Furthermore, we have demonstrated that forced increase in Cx43 expression, accomplished by Cx43 overexpression, leads to enhanced gap junction plaques between cells, normalized conduction properties in these cells and stabilized intercellular communication [17]. Despite the spontaneous electrical and contractile activity of iPSC-CMs, our present data demonstrate that these cells are able to adapt to a given stimulation frequency of up to 4 Hz over long periods of time, thereby overriding their own intrinsic electrical rhythm without any appearance of irregular beats. In addition, Cx43 expression seems highly sensitive to electrical stimulation in a frequency-dependent way in this cardiomyocyte model, showing that high stimulation frequencies lead to substantial reduction in global Cx43 expression. Therefore, iPSC-CMs represent an ideal model to study the molecular control of Cx43 expression with the purpose to identify appropriate targets to restore Cx43 expression also in diseased cardiomyocytes.

### 3.2. Molecular Mechanisms of Tachypacing-Induced Cell Stress

So far, only few studies have addressed the molecular mechanisms underlying tachycardia-induced remodeling processes at the level of expression of Cx43 and other ion channels. Regarding short-term remodeling, cytosolic Ca^2+^ overload due to enhanced Ca^2+^ current during rapid stimulation has been suggested as an early trigger of AF induction [36,37]. In contrast, long-term remodeling processes induced by chronic tachypacing involve modifications of the transcription regulation and post-transcriptional modulation of the mRNA of target proteins leading to altered expression of different channel proteins. As a putative modulator of transcriptional activity, pacing-induced oxidative cell stress has been suggested. Previous studies have shown that electrical stimulation induces transforming growth factor beta (TGF-β) expression and upregulation of cardiac-specific NADPH oxidases (NOX-2 and NOX-4) probably brought about by the periodic mechanical stretching of the sarcolemma during the contractile cycle [21]. High levels of TGF-β as well as activation of both NOX-2 and NOX-4 have also been associated with cardiac disease, in particular with AF [38]. Tachypacing-induced enhanced expression of NADPH oxidases generates ROS—either superoxide anions (NOX-2-containing membrane-bound NADPH oxidase) or H_2_O_2_ (NOX-4)—and leads to oxidative distress in cardiomyocytes [21]. The question, which of both NADPH oxidases might be primarily involved in the initiation of ROS production, remains to be elucidated. NOX-4 produces H_2_O_2_, which can be directly detected by H_2_DCF, and its expression can be found in perinuclear areas, where the increase in fluorescence signal was best detected (see Figure 3A). Therefore, NOX-4 is likely to be involved in ROS production under these experimental settings. However, this does not exclude any further contribution of NOX-2 or other ROS-producing signaling cascades. In addition, oxidative distress may be even enforced by more general metabolic stress: rapid pacing-induced contractions over a long period of time require high energy production and thus increased ATP synthesis that is rapidly consumed to sustain cell activity; this may lead to a constant buildup of superoxide anions from the overburdened respiratory chain that en route via the mitochondrial permeability transition pore to or in the cytosol give rise to secondary H_2_O_2_ or lipid hydroperoxide formation. In this study, we have shown that long periods of tachypacing lead to a strong increase in ROS formation in iPSC-CMs compared to control pacing rates. Using the ROS indicator H_2_DCF, which cannot be oxidized to the fluorescent DCF by superoxide anions, it is safe to presume that the oxidative distress has been caused by direct (NOX-4) or indirect (superoxide dismutase-1 or 2) H_2_O_2_ and possibly secondary (hydroxyl radical-mediated) lipid hydroperoxide formation. This can also be inferred from the strong inhibitory effect of ebselen, which is a GSH peroxidase 1 or 4-like scavenger of H_2_O_2_ and lipid hydroperoxides, respectively [39,40]. Prevention of this oxidative distress or ‘peroxide stress’ may preserve cardiomyocytes in a healthy metabolic state despite sustained mechanical challenge.

### 3.3. Molecular Control of Cx43 Protein Expression during Stress

ROS have many cellular targets and effects. Under normal conditions, ROS production occurs as side product of molecular signaling reactions, with positive effects on physiological processes and healthy redox signaling, and therefore has also been termed oxidative eustress [14]. Such ROS signals are usually locally confined and immediately neutralized by endogenous antioxidative enzymes such as superoxide dismutases or GSH peroxidases or used for other specific reactions such as the cysteine bonding in proteins [41]. Short-term effects of oxidative distress, however, due to overt increases in ROS in cardiomyocytes, are manifold [14]. Specifically influencing the mechanical activity of cardiac cells, such effects include direct posttranslational modifications of proteins of the excitation-contraction coupling machinery. Prolonged imbalance in the redox state of cardiomyocytes leads to instable Ca^2+^ handling and arrhythmogenic modifications [42,43]. Moreover, disorders of Ca^2+^ handling and Ca^2+^ overload influence Cx43 channel function. Ca^2+^-dependent inhibition of Cx43 gap junctional permeability leads to uncoupling of cells, which affects action potential propagation and imposes a strong risk for the development of malignant re-entry arrhythmias [44]. In addition to Cx43 forming gap junctions, ROS may also enhance lateralization of Cx43 to form hemichannels at the peri-junctional plasma membrane promoting arrhythmias, as recently described [45].

In this study, we were interested in the long-term effects of tachypacing-induced oxidative distress on Cx43 protein expression. Owed to the fact that prolonged oxidative distress may lead to upregulation of multiple micro-RNAs in the heart [46], we focused on a micro-RNA that specifically modulates Cx43 expression. This miR-1 is a striated muscle-specific micro-RNA [22] and has been shown to play an important role in pathological remodeling of the heart [47,48]. Previous studies focused on atrial arrhythmogenic effects provoked by miR-1 [49]. However, proarrhythmic ventricular effects have been unclear. In cardiac diseases, miR-1 upregulation correlates with a decrease and even a dislocation of Cx43 expression [12]. Furthermore, miR-1 has been identified as a post-transcriptional regulator of Cx43 expression by complementary binding to its mRNA. As a result, the targeted Cx43 mRNA will be degraded by the proteasomal apparatus [12,50]. In the present study, we propose the novel hypothesis that oxidative distress leads to enhanced miR-1 expression, which in turn reduces Cx43 expression, resulting in less gap junction formation and altered cell–cell coupling. Our data support this hypothesis and demonstrate that oxidative distress, induced by long-term tachypacing, strongly increases miR-1 expression in iPSC-CMs—and this effect can be effectively prevented by the GSH peroxidase mimetic ebselen. Despite abundant evidence in the literature that miR-1 expression correlates with oxidative distress, the signaling mechanisms underlying this correlation have not yet been identified. Previous studies suggest that miR-1 expression is tightly controlled by serum response factor (SRF), a highly conserved and widely expressed transcription factor regulating cell growth and cardiac development [51,52,53]. SRF binding to the promoter regions of miR-1 leads to higher miR-1 expression and consequently to enhanced degradation of Cx43 mRNA. Moreover, SRF may be closely related to cardiac disease and arrhythmia [52], raising the new question whether SRF may be a candidate for the missing link passing on the effects of chronic distress to the regulation of miR-1 expression. However, to date, these ideas remain speculative, and it will be exciting to follow up on this challenging question in future investigations.

Back to our initial hypothesis, we could demonstrate here that specific inhibition of miR-1 by an antimiR-1 antisense oligonucleotide significantly enhanced global as much as membrane-specific Cx43 expression, most likely by disinhibition of Cx43 expression via miR-1 as mentioned above. Even though the ratio of intracellular Cx43 (assembled and stored in the ER/SR and the Golgi apparatus) and its membrane expression has not yet been assessed, enhanced Cx43 expression via reduced miR-1-dependent Cx43 mRNA degradation affects both pools of Cx43, intracellular stores as well as membrane localization. While membrane shuttling and the overall turnover rate of Cx43 can be influenced by many factors including phosphorylation and other posttranslational modifications [54,55,56,57], the expression control by miR-1 is sufficiently robust to explain our experimental findings. The general increase in Cx43 expression upon miR-1 inhibition was even strong enough to lead to a remarkable functional increase in intercellular communication as assessed by the dye-diffusion experiments using FRAP. These experiments not only demonstrate that blocking miR-1 disinhibits Cx43 expression, but further prove that the new connexins assemble successfully in the membrane to produce functional gap junctions.

**Figure 7:**
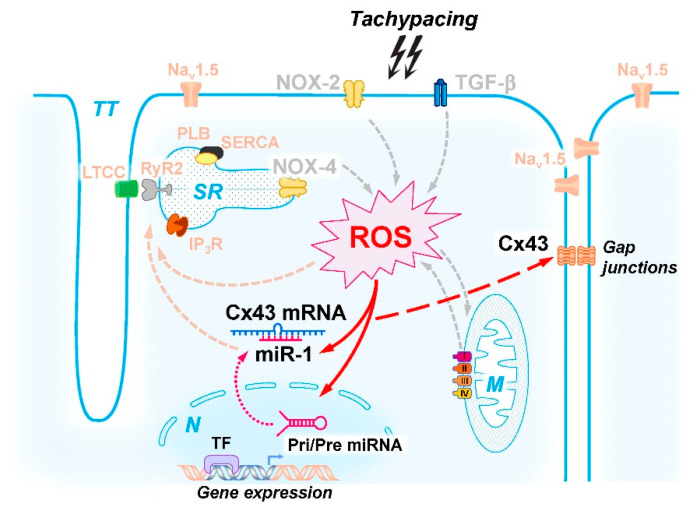
Summary of the proposed signaling cascade of chronic tachypacing-induced Cx43 expression regulation: tachy-stimulation leads to oxidative distress or peroxide stress (designated as ROS) possibly by the indicated enzymes or signaling pathways (e.g., NOX-2, NOX-4, TGF-β, mitochondria, grey arrows). The resulting increase in peroxides (H_2_O_2_, lipid hydroperoxides) triggers enhanced miR-1 expression, presumably by transcriptional upregulation of miR-1. miR-1 leads to enhanced degradation of Cx43 mRNA, resulting in reduced Cx43 protein expression and gap junction formation at the membrane. Putative parallel influences of ROS and/or miR-1 especially on the excitation-contraction coupling mechanism, are indicated in orange. *Abbreviations:* IP_3_R: IP_3_-receptor, LTCC: L-type Ca^2+^ channel, M: mitochondria, N: nucleus, Na_v_1.5: voltage-dependent Na^+^ channel, NOX-2/4: NADPH oxidases 2 and 4, PLB: phospholamban, RyR: ryanodine receptor, SERCA: SR Ca^2+^ ATPase, SR: sarcoplasmic reticulum, TGF-β: transforming growth factor beta, TT: transverse tubules.

Taken together, our data provide new mechanistic insight into the proposed signaling cascade linking high frequency electrical pacing with reduced Cx43 expression via an oxidative distress-mediated increase in miR-1 (Figure 7).

### 3.4. Implications for iPSC-CM Properties and Clinical Perspective

In light of the well-known immaturity of iPSC-CMs and the related deficiencies in electrical signal propagation across multicellular preparations, our recent findings propose a new possibility to enhance gap junction formation in these cells. It has been previously described that iPSC-CMs are in general under tight epigenetic control by miRNAs [58], which may be interpreted as a developmental trait. Therefore, reduction or neutralization of miR-1 may represent one mechanism to increase Cx43 expression and improve conduction in these cells. Apart from improved intercellular coupling, iPSC-CMs with higher Cx43 expression will also profit from increased voltage-gated sodium channel (Na_v_1.5) function, which is functionally linked to Cx43 membrane expression as previously described [17]. Together, this may lead to overall improved electrical function in terms of cell excitability and general signal spreading and therefore, better maturation of the electrical properties of iPSC-CMs.

Apart from the desired optimization of iPSC-CMs for their anticipated applications in cardiac cell replacement therapies, the proposed signaling cascade of stress-induced Cx43 remodeling elucidated in this study also proposes novel tools and targets to modulate Cx43 expression in arrhythmogenic diseases. Since the single key players investigated here play a major role in many cardiac diseases, thereby paving the way for the development of arrhythmias, it might be interesting to further explore the power of miR-1 inhibition in conditions of impaired Cx43 membrane expression. While oxidative distress in diseased hearts has been addressed by antioxidant therapies in several studies with rather unsatisfactory outcome [59], modulation of Cx43 expression at the level of miR-1-dependent degradation represents a novel idea as new target for pharmacological interventions. Indeed, there is clinical evidence that Cx43 expression is also disrupted in humans due to elevated levels of miR-1. For example, increasing levels of miR-1 have been detected in human atria in an age-dependent manner [60]. In heart failure, both decreased and increased expression levels of miR-1 have been demonstrated [61]. In another study, hearts that had to be explanted due to heart insufficiency, the expression of a large number of microRNAs, among them also miR-1, were upregulated [62]. Moreover, Cx43 expression was evaluated in explanted hearts after heart failure: these hearts showed lateralization of Cx43 and a decrease in total expression of Cx43. These molecular changes significantly reduced the conduction velocity in the diseased hearts [63].

Therefore, it will be interesting in future experiments to investigate the power of miR-1 inhibition on Cx43 expression in different models of arrhythmogenic diseases. On the other hand, our findings of the GSH peroxidase mimetic ebselen conferring strong protection against the deleterious effects of miR-1 may be equally worthwhile to follow up.

### 3.5. Limitations of the Study

Using cutting-edge methods, we tested and verified our new hypothesis that tachypacing-induced oxidative stress leads to reduced Cx43 expression via activation of miR-1. However, one limitation of this study is that these experiments have been performed in murine iPSC-CMs. Despite the fact that the identified cellular processes occur at subcellular level and may therefore represent a general concept independent of the mammalian species, this signaling pathway will have to be confirmed in human-derived iPSC-CMs. This will be the major scope of our future studies.

## 4. Materials and Methods

A detailed description of the methods used in this project can be found in the Supplemental Information.

Experiments were performed on murine induced pluripotent stem cell-derived cardiomyocytes (iPSC-CMs, Cor.At^®^), obtained from Ncardia (Cologne, Germany), and on the murine atrial HL-1 cell line, kindly provided by the late Dr. William Claycomb (Louisiana State University School of Medicine, New Orleans, LA, USA) [64]. Cells were cultured according to the manufacturers guidelines and kept in a humidified incubator at 37 °C with 5% CO_2_ until further experimentation. After establishing the protocols for tachypacing and ROS imaging, cells were evaluated for the expression of Cx43 by RT-qPCR using the StepOnePlus (Applied Biosystems, Foster City, CA, USA) PCR system and TaqMan MicroRNA probes and primers according to the manufacturer’s protocol as previously described [65]. Western blotting and immunocytochemistry were performed using standard procedures. All uncropped Western blot images are provided in the Supplemental File. For selected experiments, Cx43 expression was enhanced by transduction using adeno-associated virus AAV1/2 containing genes for either Cx43 and the reporter gene dsRed or for control dsRed alone. miR-1 activity was modulated by treating the cells with a fluorescein-labelled power inhibitor against miR-1, called antimir-1 (purchased from Qiagen, Copenhagen, Denmark). In control experiments, scramble DNA provided by the same company, was used under the same conditions. Cells were electrically paced at different stimulation frequencies during culture over prolonged period of time (24, 48, 72 h) to provoke the generation of oxidative stress, which was assessed and quantified by confocal imaging of ROS production using 6-chloromethyl-2,7-dichlorodihydrofluorescein diacetate (CM-H_2_DCF-DA, abbreviated here as H_2_DCF, ThermoFisher Scientific, Dreieich, Germany). The use of DCF as an indicator of ROS production is critically evaluated in the literature [66]. The ROS scavenger ebselen was used to evaluate tachypacing-induced ROS production. The effect of miR-1 inhibition on intercellular coupling was functionally assessed in FRAP experiments (fluorescence recovery after photobleaching of calcein). Analysis was performed as previously described in Körner et al., and the fast time constant τ1 was taken as indicator of the diffusion rate [35].

### Statistical Analysis

Image analyses were performed in ImageJ/Fiji (open source by NIH Image) and OriginPro (OriginLab Corporation, versions 2018-2021, Northampton, MA, USA). Data are presented as means ± standard error of the mean (SEM), where n corresponds to the number of repeated experiments. Statistical differences were determined by ANOVA and Student’s *t*-test where appropriate and considered as statistically significantly different at *p* < 0.05. Significant differences in the comparison are indicated by *, while ns stands for not significantly different.

## 5. Conclusions

In summary, we present the new idea to address miR-1 as a potent regulator of Cx43 expression in conditions of chronic stress in cardiomyocytes. We report that during oxidative distress, miR-1 is significantly upregulated thereby limiting Cx43 protein synthesis. As a consequence, gap junction formation and intercellular coupling are severely compromised leading to a dysfunctional syncytium. Selective blockade of miR-1 during oxidative distress efficiently prevents inhibition of Cx43 expression. Therefore, we propose miR-1 as a new target to specifically address the maladaptive remodeling processes of gap junctions contributing to the development of arrhythmias in cardiac diseases.

## Supplementary Materials

The following supporting information can be downloaded at: https://www.mdpi.com/article/10.3390/ijms231710174/s1.

## References

[1] Strait J.B., Lakatta E.G. (2012). Aging-Associated Cardiovascular Changes and Their Relationship to Heart Failure. Heart Fail. Clin..

[2] Tribulova N., Egan Benova T., Szeiffova Bacova B., Viczenczova C., Barancik M. (2015). New aspects of pathogenesis of atrial fibrillation: Remodeling of intercalated discs. J. Physiol. Pharmacol..

[3] Steenman M., Lande G. (2017). Cardiac aging and heart disease in humans. Biophys. Rev..

[4] Gude N.A., Broughton K.M., Firouzi F., Sussman M.A. (2018). Cardiac ageing: Extrinsic and intrinsic factors in cellular renewal and senescence. Nat. Rev. Cardiol..

[5] Husti Z., Varró A., Baczkó I. (2021). Arrhythmogenic remodeling in the failing heart. Cells.

[6] Andelova K., Bacova B.S., Sykora M., Hlivak P., Barancik M., Tribulova N. (2022). Mechanisms Underlying Antiarrhythmic Properties of Cardioprotective Agents Impacting Inflammation and Oxidative Stress. Int. J. Mol. Sci..

[7] Dhein S., Salameh A. (2021). Remodeling of cardiac gap junctional cell–cell coupling. Cells.

[8] Nielsen M.S., Axelsen L.N., Sorgen P.L., Verma V., Delmar M., Holstein-Rathlou N.H. (2012). Gap junctions. Compr. Physiol..

[9] Stroemlund L.W., Jensen C.F., Qvortrup K., Delmar M., Nielsen M.S. (2015). Gap junctions—Guards of excitability. Biochem. Soc. Trans..

[10] Spach M.S., Heidlage J.F. (1995). The stochastic nature of cardiac propagation at a microscopic level: Electrical description of myocardial architecture and its application to conduction. Circ. Res..

[11] Hesketh G.G., Shah M.H., Halperin V.L., Cooke C.A., Akar F.G., Yen T.E., Kass D.A., MacHamer C.E., Van Eyk J.E., Tomaselli G.F. (2010). Ultrastructure and regulation of lateralized connexin43 in the failing heart. Circ. Res..

[12] Klotz L.O. (2012). Posttranscriptional regulation of connexin-43 expression. Arch. Biochem. Biophys..

[13] Van Wagoner D.R. (2008). Oxidative stress and inflammation in atrial fibrillation: Role in pathogenesis and potential as a therapeutic target. J. Cardiovasc. Pharmacol..

[14] Sies H. (2020). Findings in redox biology: From H_2_O_2_ to oxidative stress. J. Biol. Chem..

[15] Marcu I.C., Illaste A., Heuking P., Jaconi M.E., Ullrich N.D. (2015). Functional characterization and comparison of intercellular communication in stem cell-derived cardiomyocytes. Stem Cells.

[16] Kucera J.P., Prudat Y., Marcu I.C., Azzarito M., Ullrich N.D. (2015). Slow conduction in mixed cultured strands of primary ventricular cells and stem cell-derived cardiomyocytes. Front. Cell Dev. Biol..

[17] Sottas V., Wahl C.M., Trache M.C., Bartolf-Kopp M., Cambridge S., Hecker M., Ullrich N.D. (2018). Improving electrical properties of iPSC-cardiomyocytes by enhancing Cx43 expression. J. Mol. Cell. Cardiol..

[18] Shi Y., Ducharme A., Li D., Gaspo R., Nattel S., Tardif J.C. (2001). Remodeling of atrial dimensions and emptying function in canine models of atrial fibrillation. Cardiovasc. Res..

[19] Citerni C., Kirchhoff J., Olsen L.H., Sattler S.M., Gentilini F., Forni M., Zannoni A., Grunnet M., Edvardsson N., Bentzen B.H. (2020). Characterization of Atrial and Ventricular Structural Remodeling in a Porcine Model of Atrial Fibrillation Induced by Atrial Tachypacing. Front. Vet. Sci..

[20] Fenner M.F., Carstensen H., Dalgas Nissen S., Melis Hesselkilde E., Scott Lunddahl C., Adler Hess Jensen M., Loft-Andersen A.V., Sattler S.M., Platonov P., El-Haou S. (2020). Effect of selective IK, ACh inhibition by XAF-1407 in an equine model of tachypacing-induced persistent atrial fibrillation. Br. J. Pharmacol..

[21] Yeh Y.H., Kuo C.T., Chan T.H., Chang G.J., Qi X.Y., Tsai F., Nattel S., Chen W.J. (2011). Transforming growth factor-β and oxidative stress mediate tachycardia-induced cellular remodelling in cultured atrial-derived myocytes. Cardiovasc. Res..

[22] Townley-Tilson W.H.D., Callis T.E., Wang D. (2010). MicroRNAs 1, 133, and 206: Critical factors of skeletal and cardiac muscle development, function, and disease. Int. J. Biochem. Cell Biol..

[23] Su X., Liang H., Wang H., Chen G., Jiang H., Wu Q., Liu T., Liu Q., Yu T., Gu Y. (2017). Over-expression of microRNA-1 causes arrhythmia by disturbing intracellular trafficking system. Sci. Rep..

[24] Zhang Y., Sun L., Zhang Y., Liang H., Li X., Cai R., Wang L., Du W., Zhang R., Li J. (2013). Overexpression of microRNA-1 causes atrioventricular block in rodents. Int. J. Biol. Sci..

[25] Liu Q., Zhao X., Peng R., Wang M., Zhao W., Gui Y.J., Liao C.X., Xu D.Y. (2017). Soluble epoxide hydrolase inhibitors might prevent ischemic arrhythmias via microRNA-1 repression in primary neonatal mouse ventricular myocytes. Mol. Biosyst..

[26] Yang C., Jiang L., Zhang H., Shimoda L.A., Deberardinis R.J., Semenza G.L. (2014). Analysis of hypoxia-induced metabolic reprogramming. Methods Enzymol..

[27] Puranam K.L., Laird D.W., Revel J.P. (1993). Trapping an intermediate form of connexin43 in the Golgi. Exp. Cell Res..

[28] Nattel S., Li D. (2000). Ionic remodeling in the heart: Pathophysiological significance and new therapeutic opportunities for atrial fibrillation. Circ. Res..

[29] Hanna N., Cardin S., Leung T.K., Nattel S. (2004). Differences in atrial versus ventricular remodeling in dogs with ventricular tachypacing-induced congestive heart failure. Cardiovasc. Res..

[30] Akar F.G., Spragg D.D., Tunin R.S., Kass D.A., Tomaselli G.F. (2004). Mechanisms underlying conduction slowing and arrhythmogenesis in nonischemic dilated cardiomyopathy. Circ. Res..

[31] Akar F.G., Nass R.D., Hahn S., Cingolani E., Shah M., Hesketh G.G., DiSilvestre D., Tunin R.S., Kass D.A., Tomaselli G.F. (2007). Dynamic changes in conduction velocity and gap junction properties during development of pacing-induced heart failure. Am. J. Physiol. Heart Circ. Physiol..

[32] Sun H., Gaspo R., Leblanc N., Nattel S. (1998). Cellular mechanisms of atrial contractile dysfunction caused by sustained atrial tachycardia. Circulation.

[33] Meraviglia V., Azzimato V., Colussi C., Florio M.C., Binda A., Panariti A., Qanud K., Suffredini S., Gennaccaro L., Miragoli M. (2015). Acetylation mediates Cx43 reduction caused by electrical stimulation. J. Mol. Cell. Cardiol..

[34] Silbernagel N., Körner A., Balitzki J., Jaggy M., Bertels S., Richter B., Hippler M., Hellwig A., Hecker M., Bastmeyer M. (2020). Shaping the heart: Structural and functional maturation of iPSC-cardiomyocytes in 3D-micro-scaffolds. Biomaterials.

[35] Körner A., Mosqueira M., Hecker M., Ullrich N.D. (2021). Substrate Stiffness Influences Structural and Functional Remodeling in Induced Pluripotent Stem Cell-Derived Cardiomyocytes. Front. Physiol..

[36] Daoud E.G., Knight B.P., Weiss R., Bahu M., Paladino W., Goyal R., Man K.C., Strickberger S.A., Morady F. (1997). Effect of verapamil and procainamide on atrial fibrillation-induced electrical remodeling in humans. Circulation.

[37] Yu W.C., Chen S.A., Lee S.H., Tai C.T., Feng A.N., Kuo B.I.T., Ding Y.A., Chang M.S. (1998). Tachycardia-induced change of atrial refractory period in humans: Rate dependency and effects of antiarrhythmic drugs. Circulation.

[38] Reilly S.N., Jayaram R., Nahar K., Antoniades C., Verheule S., Channon K.M., Alp N.J., Schotten U., Casadei B. (2011). Atrial sources of reactive oxygen species vary with the duration and substrate of atrial fibrillation: Implications for the antiarrhythmic effect of statins. Circulation.

[39] Müller A., Cadenas E., Graf P., Sies H. (1984). A novel biologically active seleno-organic compound-1. Glutathione peroxidase-like activity in vitro and antioxidant capacity of PZ 51 (Ebselen). Biochem. Pharmacol..

[40] Santi C., Scimmi C., Sancineto L. (2021). Ebselen and analogues: Pharmacological properties and synthetic strategies for their preparation. Molecules.

[41] Neuman R.B., Bloom H.L., Shukrullah I., Darrow L.A., Kleinbaum D., Jones D.P., Dudley S.C. (2007). Oxidative Stress Markers Are Associated with Persistent Atrial Fibrillation. Clin. Chem..

[42] Saraf A., Rampoldi A., Chao M., Li D., Armand L., Hwang H., Liu R., Jha R., Fu H., Maxwell J.T. (2021). Functional and molecular effects of TNF-α on human iPSC-derived cardiomyocytes. Stem Cell Res..

[43] Weissman D., Maack C. (2021). Redox signaling in heart failure and therapeutic implications. Free Radic. Biol. Med..

[44] Tribulova N., Knezl V., Szeiffova Bacova B., Egan Benova T., Viczenczova C., Gonçalvesova E., Slezak J. (2016). Disordered myocardial Ca^2+^ homeostasis results in substructural alterations that may promote occurrence of malignant arrhythmias. Physiol. Res..

[45] Andelova K., Benova T.E., Bacova B.S., Sykora M., Prado N.J., Diez E.R., Hlivak P., Tribulova N. (2021). Cardiac connexin-43 hemichannels and pannexin1 channels: Provocative antiarrhythmic targets. Int. J. Mol. Sci..

[46] Climent M., Viggiani G., Chen Y.W., Coulis G., Castaldi A. (2020). Microrna and ros crosstalk in cardiac and pulmonary diseases. Int. J. Mol. Sci..

[47] Chistiakov D.A., Orekhov A.N., Bobryshev Y.V. (2016). Cardiac-specific miRNA in cardiogenesis, heart function, and cardiac pathology (with focus on myocardial infarction). J. Mol. Cell. Cardiol..

[48] Santulli G., Iaccarino G., De Luca N., Trimarco B., Condorelli G. (2014). Atrial fibrillation and microRNAs. Front. Physiol..

[49] Girmatsion Z., Biliczki P., Bonauer A., Wimmer-Greinecker G., Scherer M., Moritz A., Bukowska A., Goette A., Nattel S., Hohnloser S.H. (2009). Changes in microRNA-1 expression and IK1 up-regulation in human atrial fibrillation. Heart Rhythm.

[50] Stein C.A., Hansen J.B., Lai J., Wu S.J., Voskresenskiy A., Høg A., Worm J., Hedtjärn M., Souleimanian N., Miller P. (2009). Efficient gene silencing by delivery of locked nucleic acid antisense oligonucleotides, unassisted by transfection reagents. Nucleic Acids Res..

[51] Chen Y., Liu Q., Yang T., Shen L., Xu D. (2021). Soluble Epoxide Hydrolase Inhibitors Regulate Ischemic Arrhythmia by Targeting MicroRNA-1. Front. Physiol..

[52] Lu Y., Zhang Y., Shan H., Pan Z., Li X., Li B., Xu C., Zhang B., Zhang F., Dong D. (2009). MicroRNA-1 downregulation by propranolol in a rat model of myocardial infarction: A new mechanism for ischaemic cardioprotection. Cardiovasc. Res..

[53] Zhao Y., Samal E., Srivastava D. (2005). Serum response factor regulates a muscle-specific microRNA that targets Hand2 during cardiogenesis. Nature.

[54] Smyth J.W., Hong T.T., Gao D., Vogan J.M., Jensen B.C., Fong T.S., Simpson P.C., Stainier D.Y.R., Chi N.C., Shaw R.M. (2010). Limited forward trafficking of connexin 43 reduces cell-cell coupling in stressed human and mouse myocardium. J. Clin. Investig..

[55] Laird D.W., Castillo M., Kasprzak L. (1995). Gap junction turnover, intracellular trafficking, and phosphorylation of connexin43 in brefeldin A-treated rat mammary tumor cells. J. Cell Biol..

[56] Qin H., Shao Q., Igdoura S.A., Alaoui-Jamali M.A., Laird D.W. (2003). Lysosomal and proteasomal degradation play distinct roles in the life cycle of Cx43 in gap junctional intercellular communication-deficient and -competent breast tumor cells. J. Biol. Chem..

[57] Thomas T., Jordan K., Simek J., Shao Q., Jedeszko C., Walton P., Laird D.W. (2005). Mechanism of Cx43 and Cx26 transport to the plasma membrane and gap junction regeneration. J. Cell Sci..

[58] Li N., Long B., Han W., Yuan S., Wang K. (2017). MicroRNAs: Important regulators of stem cells. Stem Cell Res. Ther..

[59] van der Pol A., van Gilst W.H., Voors A.A., van der Meer P. (2019). Treating oxidative stress in heart failure: Past, present and future. Eur. J. Heart Fail..

[60] Biliczki P., Boon R.A., Girmatsion Z., Bukowska A., Ördög B., Kaess B.M., Hohnloser S.H., Goette A., Varró A., Moritz A. (2019). Age-related regulation and region-specific distribution of ion channel subunits promoting atrial fibrillation in human left and right atria. Europace.

[61] Topkara V.K., Mann D.L. (2011). Role of microRNAs in cardiac remodeling and heart failure. Cardiovasc. Drugs Ther..

[62] Lai K.B., Sanderson J.E., Izzat M.B., Yu C.M. (2015). Micro-RNA and mRNA myocardial tissue expression in biopsy specimen from patients with heart failure. Int. J. Cardiol..

[63] Glukhov A.V., Fedorov V.V., Kalish P.W., Ravikumar V.K., Lou Q., Janks D., Schuessler R.B., Moazami N., Efimov I.R. (2012). Conduction remodeling in human end-stage nonischemic left ventricular cardiomyopathy. Circulation.

[64] Claycomb W.C., Lanson N.A., Stallworth B.S., Egeland D.B., Delcarpio J.B., Bahinski A., Izzo N.J. (1998). HL-1 cells: A cardiac muscle cell line that contracts and retains phenotypic characteristics of the adult cardiomyocyte. Proc. Natl. Acad. Sci. USA.

[65] Schmidt C., Wiedmann F., Zhou X.B., Heijman J., Voigt N., Ratte A., Lang S., Kallenberger S.M., Campana C., Weymann A. (2017). Inverse remodelling of K2P 3.1 K+ channel expression and action potential duration in left ventricular dysfunction and atrial fibrillation: Implications for patient-specific antiarrhythmic drug therapy. Eur. Heart J..

[66] Kalyanaraman B., Darley-Usmar V., Davies K.J.A., Dennery P.A., Forman H.J., Grisham M.B., Mann G.E., Moore K., Roberts L.J., Ischiropoulos H. (2012). Measuring reactive oxygen and nitrogen species with fluorescent probes: Challenges and limitations. Free Radic. Biol. Med..

