# Supplementary material for: Distress-Mediated Remodeling of Cardiac Connexin-43 in a Novel Cell Model for Arrhythmogenic Heart Diseases
Source: Int J Mol Sci. 2022 Sep 5;23(17):10174. doi: 10.3390/ijms231710174 (PMC9456330; doi:10.3390/ijms231710174)
Supplement: Supplementary file 1 [file ijms-23-10174-s001.zip › ijms-1869106-supplementary.pdf]

Supplemental Information:
Distress-mediated remodeling of cardiac connexin-43 in a novel cell model for arrhythmogenic heart diseases

Carl-Mattheis Wahl[1], Constanze Schmidt[2,3], Markus Hecker[1,3], Nina D. Ullrich[1,3*]

[1]Institute of Physiology and Pathophysiology, Division of Cardiovascular Physiology, Heidelberg University, 69120 Heidelberg, Germany;

[2]Department of Cardiology, Angiology and Pneumology, University Hospital Heidelberg, 69120, Heidelberg, Germany;

[3]DZHK (German Center for Cardiovascular Research), Partner Site Heidelberg/Mannheim, University of Heidelberg, Heidelberg, Germany.

[*]***Correspondence to:*** Nina D. Ullrich, PhD

Supplemental Figures: uncropped Western blots of Figures 4A (Suppl. Fig. S1) and 5C (Suppl. Fig. S2) of the main text:

Supplemental Figure S1 (related to Figure 4A):

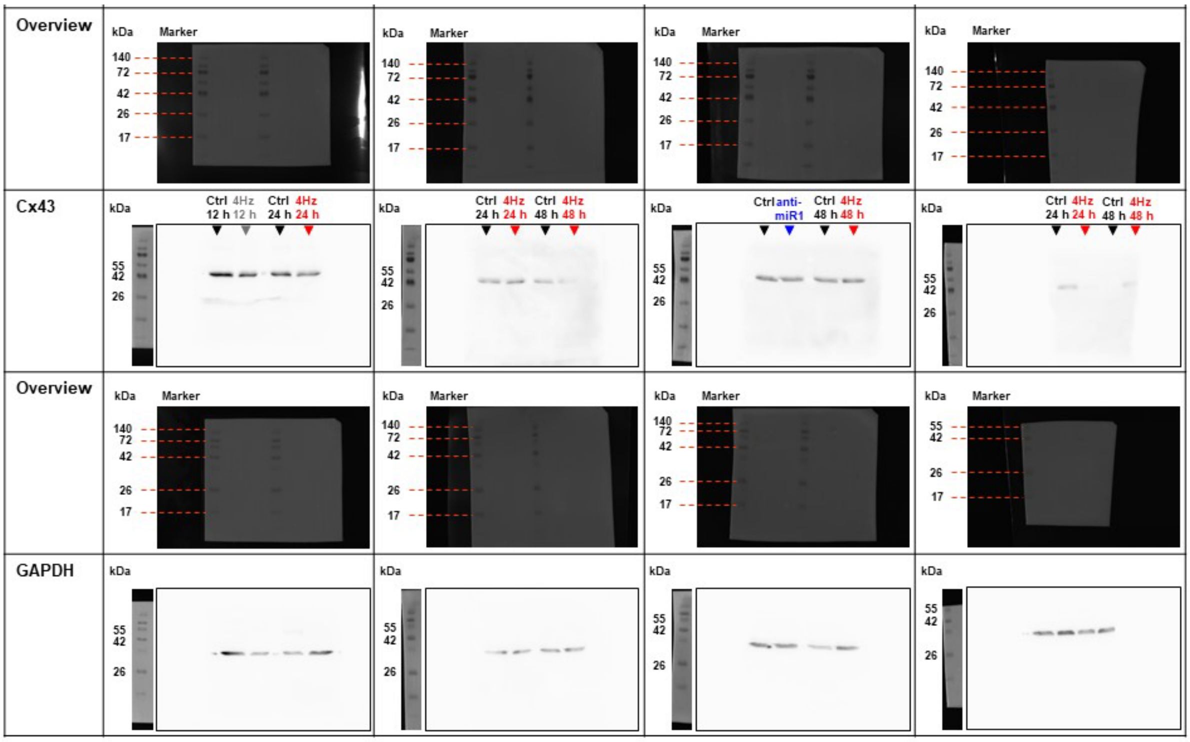

Supplemental Figure S1: Original Western blots used for the analysis of total Cx43 protein expression in iPSC-CMs under control conditions (Ctrl, paced at 1 Hz, black) and during tachypacing (4 Hz, red) for the indicated durations of 24 or 48 h. Data from the second blot from the left are shown as sample signals in **Figure 4A** of the main text (first, second and fourth lanes). Results of 12 h served as initial test and were not quantified. The overview images show the picture of the blot displaying the marker staining to identify protein sizes (in kDa). The lower images show either Cx43 or GAPDH immunolabeling. GAPDH served as internal loading control and was used to normalize the Cx43 signal

to its relative expression level. *Please note:* the left 2 lanes in the third blot from the left (Ctrl and antimiR1) belong to the results of Supplemental Figure S2.

Supplemental Figure S2 (related to Figure 5C):

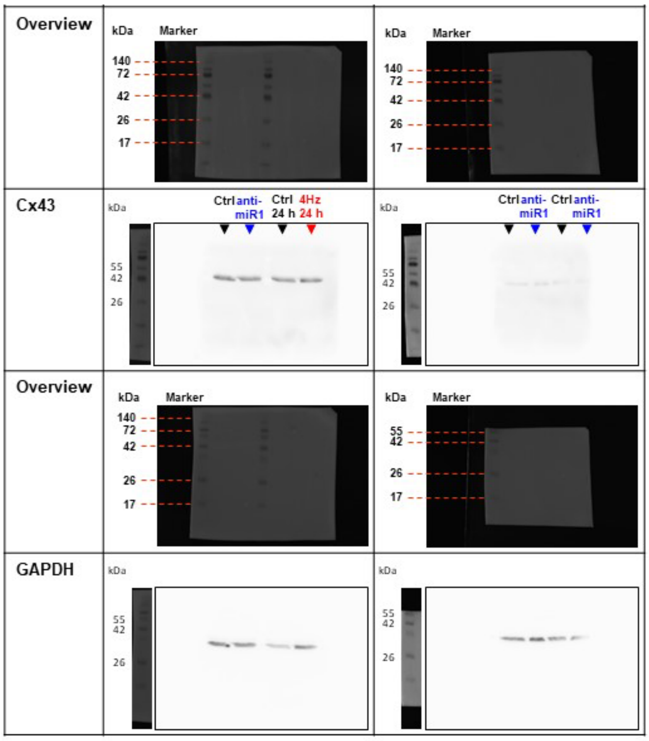

Supplemental Figure S2: Original Western blots used for the analysis of total Cx43 protein expression in iPSC-CMs without (Ctrl, black) and after treatment with the oligonucleotide antimiR1 (blue). Again, the overview images show the marker staining to identify protein sizes (in kDa). The images underneath show either Cx43 or GAPDH (as loading control) immunolabeling. The first 2 lanes from the left blot are shown as sample signals in **Figure 5C** of the main text. *Please note:* the right 2 lanes in the left blot (Ctrl 24 h and 4 Hz 24 h) belong to the results of Supplemental Figure S1.

Supplemental experimental procedures:

General methods

Cardiac cell culture models

Murine induced pluripotent stem cell-derived cardiomyocytes (iPSC-CMs, Cor.At®) were obtained from Ncardia (Cologne, Germany). Frozen stocks were kept in liquid nitrogen until use. After thawing, cells were seeded at 90% confluency on custom-built 35 mm Lumox®-dishes (Lumox foil from Sarstedt, Germany) for functional experiments (pacing, Ca^{2+} imaging, ROS-imaging and fluorescence recovery after photobleaching, FRAP). For Western blot and RT-qPCR experiments, cells were seeded at 90% confluency on 35 mm plastic dishes (Corning, Sigma-Aldrich, Germany). All culture dishes were coated with a mixture of fibronectin and laminin in PBS (1:1:100) to ensure cell attachment after seeding. Cells were kept in Cor.At® culture medium.

The murine atrial HL-1 cell line was kindly provided by the late Dr. William Claycomb (Louisiana State University School of Medicine, New Orleans, USA)[18]. HL-1 cells were cultured on gelatin-fibronectin coated glass-bottom dishes (35 mm, MatTech, USA) in growth medium composed of Claycomb medium, 10% FBS, 100 µM norepinephrine, 2 mM L-glutamine and 1% penicillin/streptomycin.

All cells were kept in a humidified incubator at 37°C with 5% CO_2.

Molecular biology and protein biochemistry

Modulation of miR-1

iPSC-CMs were treated by direct addition of either 25, 50 or 100 nM of phosphorothioate-modified, 5‘-fluorescein labeled power inhibitors against mature miR-1 (power-locked-nucleic-acid, LNA, 5′ -ACATACTTCTTTACATTCCA-3′; Qiagen, Denmark), termed antimiR-1, to the cell culture medium. Control cells were treated with the corresponding scramble DNA (5′-TAACACGTCTATACGCCCA-3′). After 24 h, the cell culture medium was refreshed. Expression efficiency was monitored by daily control of the FITC fluorescence by fluorescence microscopy. Cells were processed for further experiments after 72 h.

Transduction

iPSC-CMs were transduced with adeno-associated viruses containing genes for either connexin-43 and the reporter gene dsRed coupled via IRES or the reporter gene dsRed alone for control ($AAV_{1/2}$-Cx43-IRES-DsRED-Express2 or $AAV_{1/2}$-DsRED-Express2, 0.5 µl/ml medium). Gene expression was monitored by the expression of dsRed and usually started after 4-5 days after transduction at an efficiency of ~95%.

Western blotting

Cells were lysed in lysis buffer composed of 50 mM Tris pH 7.5, 150 mM NaCl, 4% complete protease inhibitor (Merck, Darmstadt, Germany), 0.25% SDS, 1% NP40 for 30 min at 4°C on a rotator wheel. The lysate was centrifuged for 15 min at 12.000 g at 4°C and the supernatant was further processed. Protein quantification was performed using Bradford staining. 30 µg of proteins were loaded on a 12% SDS-acrylamide gel. Transfer was performed for 40 min on a PVDF-membrane. The membrane was probed with antibodies against Cx43 (Millipore, MAB3067, 1:500 in TBS-Tween, 5% milk) and GAPDH (Life Technology, MA5-15738, 1:500 in TBS-Tween, 5% milk) at 4 °C overnight. After washing with TBS-Tween, secondary antibody (goat IgG-horseradish peroxidase, 1:5000 in TBS, 5% milk) was incubated for 1 h at RT. For development, the membrane was incubated with Luminata Forte (Merck, Darmstadt, Germany) for 3 min at RT. Images were developed on ImageQuant LAS 4000 software. Densities were analyzed in ImageJ/Fiji (open source by NIH Image). All uncropped Western blot images are provided in the Supplemental File.

RT-qPCR

RNA isolation from cultured iPSC-CMs was done using TRIzol Reagent (Invitrogen, Thermo Fisher Scientific, Waltham, MA, USA) according to the manufacturer’s instructions. For reverse transcription of miRNA into complementary DNA (cDNA) the TaqMan microRNA Kit for reverse transcription (Applied Biosystems, Foster City, California, United States) and specific RT primers (TaqMan MicroRNA Assay, Applied Biosystems) were used. Reverse transcription of total RNA was performed using the Maxima First Strand cDNA Synthesis Kit (Thermo Scientific). Quantitative real-time polymerase chain reaction (qPCR) experiments were performed using the StepOnePlus (Applied Biosystems) PCR system and TaqMan MicroRNA probes and primers according to the manufacturer’s protocol as described[19]. All qPCR reactions, as well as control experiments without cDNA, were performed in triplicates.

Measurements of miRNA expression were normalized by the geometric mean of miR 26b, U47, and RNU6B expression.

Immunocytochemistry

Cells were grown on glass coverslips, washed in PBS and fixed in 4% para-formaldehyde (PFA, ThermoFisher Scientific, Dreieich, Germany) for 10 min. After fixation, cells were washed and incubated in blocking buffer containing PBS, 1% BSA and 0.3% Triton-X100 for 30 min at RT. Cells were incubated with a primary antibody against Cx43 (Millipore, MAB3067, 1:400 diluted in blocking buffer) for 2 h at RT. After washing, cells were incubated with the secondary antibody (AlexaFluor-568 donkey-anti-mouse, 1:1000) for 1 h at RT. Glass coverslips were mounted on objective slides using Fluoroshield containing DAPI (Sigma-Aldrich, Germany) and imaged on a laser-scanning confocal microscope (LSCM, Leica TCS SP8, Mannheim, Germany). Relative fluorescence intensities were analyzed in ImageJ/Fiji (NIH Image) and OriginPro (OriginLab Corporation, Northampton, MA, USA).

Electrical pacing

Prewarmed (37°C) iPSC-CMs culture medium was changed prior to experiments. 35 mm falcon dishes were mounted into a 6-well C-dish containing pairs of platinum electrodes for each well (IonOptix, Dublin, Ireland). The C-dish was connected to a MyoPacer Field Stimulator (IonOptix, Dublin, Ireland). iPSC-CMs were exposed to bipolar pulses during culture in the incubator with a frequency of 1 Hz (control) or 4 Hz (tachypacing), at a duration of 2 ms and a voltage of 40 V for 24 h and 48 h at 37°C. The adaptation of the cells to the stimulation frequency was monitored by the video capture function of a cell culture microscope (Leica Microsystems, Mannheim, Germany). For evaluation, Ca^{2+} transients and contractions were simultaneously measured at the IonOptix setup or imaged in the line scan mode at a confocal microscope (Leica SP8).

Ca^{2+} imaging

IonOptix

iPSC-CMs were washed in Tyrode solution (composed of (in mM): NaCl 140, KCl 5.4, $MgCl_2$ 1, $CaCl_2$ 1.8, HEPES 5, Glucose 10, pH 7.4). After washing, cells were incubated with fura-2-AM (2 µM in Tyrode solution, F1221, Thermo Fisher Scientific, Darmstadt, Germany) for 20 min at RT. Afterwards, cells were kept for another 10 min to ensure complete de-esterification of fura-2. Ca^{2+} transients and contractions were monitored using the IonOptix Myocyte Calcium and Contractility System, equipped with a custom-built solution exchanger, and analyzed by the software IonWizard (IonOptix, Westwood, USA).

Confocal line scans

iPSC-CMs were washed in Tyrode solution at 37°C, then incubated with fluo-4-AM (5 µM in Tyrode solution, F14201, Thermo Fisher Scientific, Darmstadt, Germany) for 20 min plus 10 min de-esterification at 37°C. Images were recorded on a LSCM (Leica TCS SP8, Leica Microsystems, Mannheim). Confocal line scans were captured at 600 Hz scanning speed with a resolution of 512x1 pixels using a 40x magnification objective, NA 1.1. Fluo-4 was excited at 488 nm, emission was captured between 495-600 nm. Relative fluorescence intensities ($\Delta F/F_0$) were analyzed in ImageJ/Fiji (NIH Image).

ROS imaging

Evaluation of 6-chloromethyl-2',7'-dichlorodihydrofluorescein (CM-H_2DCF) as ROS indicator

Cells were washed with prewarmed Tyrode solution, composed of (in mM): NaCl 140, KCl 5.4, $MgCl_2$ 1.1, HEPES 5, Glucose 10, $CaCl_2$ 1.8, pH 7.4, then loaded with 6-chloromethyl-2,7-dichlorodihydrofluorescein diacetate (CM-H_2DCF-DA, abbreviated here as H_2DCF, 5 µM diluted in Tyrode solution, ThermoFisher Scientific, Germany) for 20 min at 37°C. The use of DCF is critically evaluated in the literature[20] . After washing and de-esterification, cells were continuously superfused with prewarmed Tyrode solution. The mild oxidant tert-Butylhydroperoxid (TBHP, 100 µM, 814006, Merck, Darmstadt, Germany) was applied to test and evaluate the ROS indicator H_2DCF at different conditions. The setup was equipped with an electronic 8-valve controlled gravity perfusion system for rapid solution exchange (ALA Scientific Instruments). Confocal images were taken using an LSCM (Olympus IX81, FluoView1000, Hamburg, Germany) at a frequency of 0.1 Hz for 1000 s at a scanning speed of 2 Hz with a resolution of 512x256 pixels using a 20x magnification objective (NA 0.75). H_2DCF in the cell is rapidly oxidized by hydrogen peroxide (H_2O_2) to the highly fluorescent DCF, which was monitored over time with the excitation wavelength set to 488 nm and the emission wavelength set to 495-600 nm.

Chronic oxidative stress

iPSC-CMs were paced during culture at 4 Hz and 40 V. Control cells were paced at 1 Hz or left unpaced. Additionally, tachypaced cells were simultaneously incubated with the hydrogen peroxide / lipid hydroperoxide scavenger ebselen (1 µM diluted in cell culture medium, E3520, Sigma-Aldrich, Munich, Germany). After 24 h, cells were washed and loaded with CM-H_2-DCF-DA. Images were taken using an LSCM (Olympus IX81, FV1000, Olympus, Hamburg, Germany). H_2DCF was excited at 488 nm, emission was captured between 495-600 nm. Single images were taken at 5 different spots in every dish at a scanning speed of 2 Hz with a resolution of 512x512 pixels using a 20x magnification objective. Under the same conditions, image series were captured at a frequency of 0.1 Hz for 50 s.

Fluorescence recovery after photobleaching (FRAP)

To evaluate intercellular coupling via gap junctions, cells were loaded with the fluorescent gap junction-permeant dye calcein (0.5 µM calcein-AM, diluted in Tyrode solution, ThermoFisher Scientific, Dreieich, Germany). Photobleaching and imaging were performed on the Olympus LSCM using a 60x water immersion objective (NA 1.2). After dye loading and de-esterification, cells were imaged. Photobleaching of one cell within a cell cluster was induced with a laser power of 50% at a scanning rate of 10 µs/pixel for 5 s. Afterwards, fluorescence recovery was resumed at the usual laser power of 0.5-1.5% at 2 ms/pixel. In total, 52 images were taken every 10 s to monitor FRAP. For analysis, background was subtracted to the point of bleaching. Then the traces were normalized to the initially recorded fluorescence intensity before bleaching. Traces were fitted with a biexponential function using OriginPro software as described in Körner *et al.*, and the fast time constant $\tau 1$ was taken as indicator of the diffusion rate[21].

Statistical analysis

Images were analyzed in ImageJ/Fiji and OriginPro. Data are displayed as change in relative fluorescence (F/F0). Data are presented as means ± standard error of the mean (SEM), where n corresponds to the number of repeated experiments. Statistical differences were determined by ANOVA and Student´s t-test where appropriate and considered as statistically significantly different at $P<0.05$. Significant differences in the comparison are indicated by *, while ns stands for not significantly different.
